# Multi-Omics Reveals Salt Stress Effects on Quality Formation of Strong-Gluten Wheat

**DOI:** 10.3390/ijms27073013

**Published:** 2026-03-26

**Authors:** Wei Zhou, Jianchao Zheng, Yonggang Zhao, Shikui Li, Hongxia Zhang, Xiang Li, Chuan Zhong, Xinglong Dai

**Affiliations:** 1 State Key Laboratory of Wheat Improvement, College of Agriculture, Shandong Agricultural University, Tai’an 271018, China; zhouwei_sdau@163.com; 2 Agricultural Science Research Institute of the Twelves Division of Xinjiang Production and Construction Crops, Urumqi 830500, China; zgxjzjc@126.com (J.Z.); 15099529923@163.com (S.L.); 3 Agricultural Science Research Institute of the Seventh Division of Xinjiang Production and Construction Crops, Yili 833200, China; nks_zyg@163.com (Y.Z.); 18893652080@163.com (H.Z.); 4 College of Life Sciences, Shandong Agricultural University, Tai’an 271018, China; lixiang@sdau.edu.cn

**Keywords:** strong-gluten wheat, salt stress, multi-omics, quality

## Abstract

Salt stress is a critical abiotic constraint affecting wheat yield and quality. In this study, we employed pot experiments under controlled salinity (2.8‰ NaCl) and multi-omics approaches to elucidate the regulatory mechanisms underlying grain quality formation in a strong-gluten wheat variety, Jinan 17. Key findings revealed that salt stress caused a significant 41.27% reduction in 1000-kernel weight, while protein content increased by 13.82%. However, bread volume and bread score were reduced by 16.85% and 13.08%, respectively. Multi-omics integration uncovered that salt stress repressed the expression of starch synthesis-related genes (e.g., *TraesCS2A03G0349200*), diverting carbon skeletons toward amino acid metabolism pathways. This metabolic reprogramming disrupted the glutenin/gliadin ratio (down 14.35%), with high molecular weight glutenin subunits (HMW-GS) synthesis being suppressed, while low molecular weight glutenin subunits (LMW-GS) and gliadin accumulated by 19.28% and 24.76%, respectively, forming a “high extensibility but low elasticity” gluten network. Furthermore, transcriptomic analysis identified significant upregulation of arginine metabolism genes (e.g., *TraesCS6A03G0029900*), which enhanced osmolyte biosynthesis and exacerbated carbon–nitrogen partitioning imbalances. This study provides novel insights into the molecular mechanisms of flour quality deterioration under saline conditions and identifies critical regulatory nodes for simultaneous improvement of starch synthesis and gluten network architecture in salt-affected wheat breeding programs.

## 1. Introduction

Wheat (*Triticum aestivum* L.) is a globally important staple food crop. Strong-gluten wheat is highly valued for its high protein content and superior gluten strength, making it particularly suitable for producing bread and other food products [1]. With the continuous improvement of living standards, the demand for high-quality, strong-gluten wheat suitable for bread production is growing. Soil salinization is a widespread and increasingly severe environmental issue that significantly reduces global crop yield and adversely affects grain quality [2,3,4]. It is estimated that more than 20% of irrigated agricultural land is affected by salinity, resulting in substantial economic losses [5]. In existing studies, elevated soil salinity reduces soil water potential, thereby impairing water uptake by wheat roots and causing water deficit [6]. This immediately suppresses leaf expansion, reduces tiller number and leaf area, and ultimately leads to a substantial decline in biomass and grain yield [7,8]. Although the adverse effects of salt stress on wheat growth, physiology, and yield have been widely reported, its specific and profound impacts on key biochemical processes governing protein quality formation remain underexplored, especially in strong-gluten varieties [9,10].

The baking quality of wheat flour is primarily determined by the quantity and composition of seed storage proteins, namely glutenin and gliadin. The unique viscoelasticity of gluten, essential for dough strength and gas retention, is largely influenced by the ratio of high molecular weight glutenin subunits (HMW-GS) to low molecular weight glutenin subunits (LMW-GS), the glutenin-to-gliadin ratio, and overall protein content [11,12]; these traits are governed by complex regulatory networks involving transcription factors, protein assembly mechanisms, and post-translational modifications, which are further modulated by abiotic stress factors [13].

Recent advances in integrated multi-omics technologies have provided new perspectives for systematically elucidating the molecular mechanisms underlying quality formation in wheat in response to abiotic stress [14,15,16]. Transcriptomic studies reveal that salt stress activates antioxidant pathways via transcription factors (e.g., MYB and WRKY) while suppressing starch biosynthesis genes such as TaGBSSI [17,18]. Proteomic analyses demonstrate reduced HMW-GS and upregulated heat shock proteins in salt-sensitive genotypes [19]. Metabolomic profiling further indicates that excessive accumulation of osmolytes (proline and betaine) may inhibit starch synthesis [20]. Multi-omics correlation analyses identified critical genes like TaSRO1, which coordinates ROS homeostasis and hormone signaling pathways, thereby simultaneously influencing salt tolerance and yield [21]. However, current omics studies investigating the effects of salt stress on wheat growth and development have predominantly focused on stress tolerance mechanisms, while research elucidating how salt stress impacts wheat protein quality, particularly in strong-gluten wheat varieties, remains limited.

Therefore, this study aims to elucidate the effects of salt stress on key protein quality parameters, including protein content, gliadin, glutenin, HMW-GS, LMW-GS, and the glutenin-to-gliadin ratio, in strong-gluten wheat. By integrating metabolomic, proteomic, and transcriptomic analyses, the aim was to uncover the molecular mechanisms governing these changes. The findings are expected to provide potential molecular targets for improving protein quality in strong-gluten wheat under salt stress conditions.

## 2. Results

### 2.1. Yield and Yield Components of Strong-Gluten Wheat

**Figure 1:**
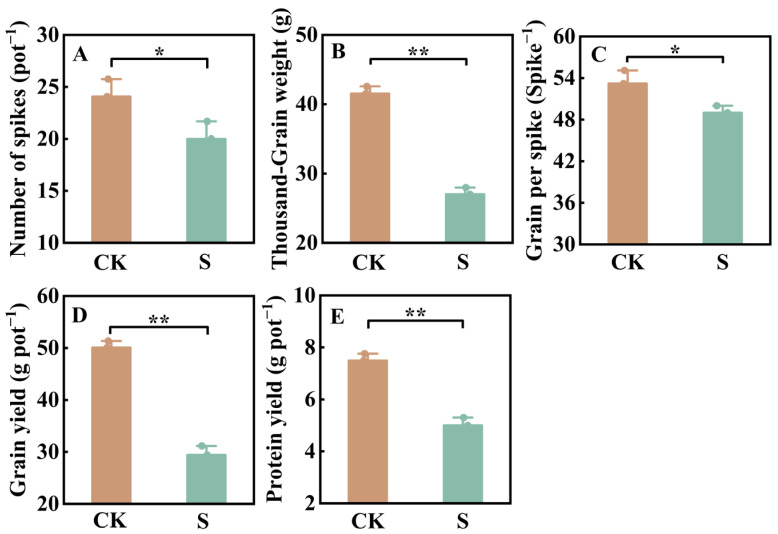
Grain yield, yield components, and protein yield. CK: Control group, S: NaCl concentration of 2.8 ‰. (**A**): Number of spikers. (**B**): Thousand-Grain weight. (**C**): Grain per spike. (**D**): Grain yield. (**E**): Protein yield. Error bars indicate the standard deviations. The points on the bar graph represent the replicate values in the processed data, and the number of samples used for each bar in the bar chart is *n* = 3. The data were analyzed by one-way ANOVA; * represents *p* < 0.05, and ** represents *p* < 0.01.

Salt stress significantly affected the single-pot yield of JN17 (*p* < 0.05). Under salt stress, the yield of JN17 decreased by 41.27% compared to the control (Figure 1D). Concurrently, salt stress also reduced the number of spikes per pot, kernels per spike, and 1000-grain weight by 16.91%, 7.94%, and 35.01%, respectively, compared to the control (Figure 1A–C), with the 1000-grain weight exhibiting the greatest reduction. These results indicate that salt stress primarily reduced wheat grain yield by decreasing the 1000-grain weight of JN17.

### 2.2. Protein Content and Composition in Strong-Gluten Wheat

**Figure 2:**
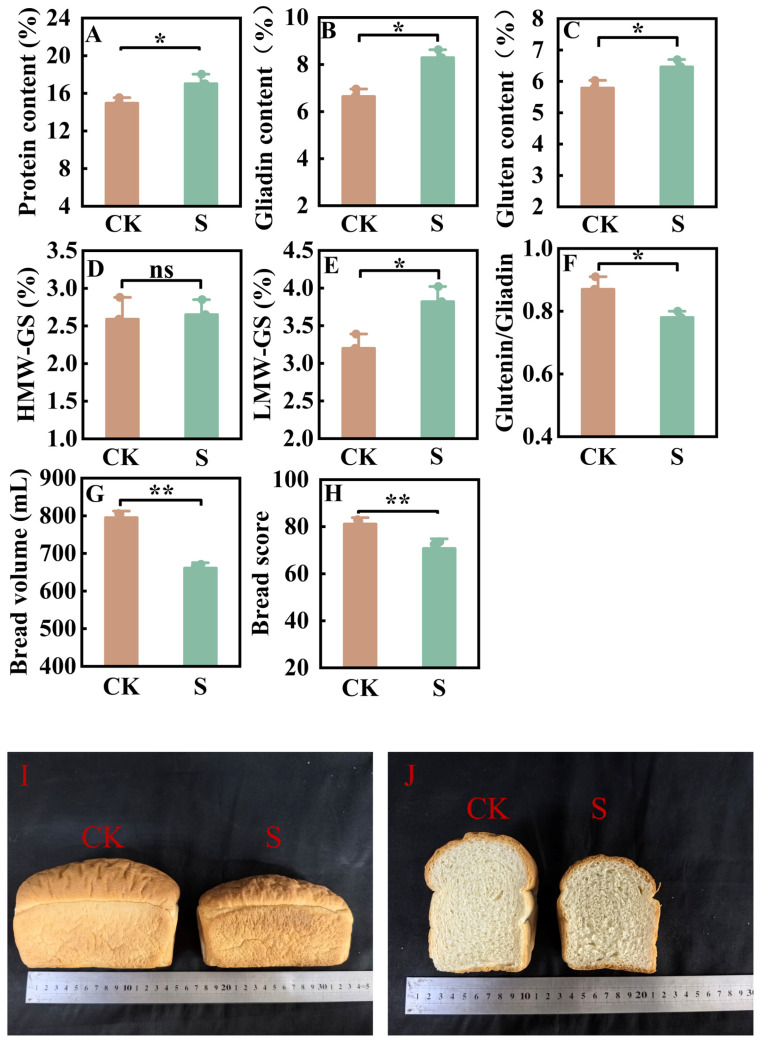
Grain protein content, quality traits, bread volume and score, and the images of bread side view and cross-section under different treatments. HMW-GS: High molecular weight glutenin subunits; LMW-GS: Low molecular weight glutenin subunits. (**A**): Protein content. (**B**): Gliadin content. (**C**): Gluten content. (**D**): HMW-GS content. (**E**): LMW-GS content. (**F**): Glutenin/Gliadin ratio. (**G**): Bread volume. (**H**): Bread score. (**I**): The bread side view of strong-gluten wheat. (**J**): The bread cross-section of strong-gluten wheat. * represents *p* < 0.05, ** represents *p* < 0.01, ns represents not significant.

Compared with the control, salt stress significantly increased the grain protein content of JN17 by 13.82% (Figure 2A). Under salt stress, the grain protein yield of JN17 decreased by 33.15%. This may be because salt stress reduced wheat yield (41.27%, Figure 1D). High-performance liquid chromatography analysis revealed that salt stress elevated the contents of glutenin and gliadin by 11.63% and 24.76%, respectively, in JN17 grains (Figure 2B,C). Further examination of glutenin subunits showed a 2.16% increase in HMW-GS and a 19.28% rise in LMW-GS under salt stress (Figure 2D,E), indicating that LMW-GS accumulation predominantly drove the glutenin enhancement. Concurrently, the glutenin-to-gliadin ratio decreased by 14.35% due to the smaller increase in glutenin content compared to that of gliadin (Figure 2F). Studies have demonstrated that salt stress enhances the protein content in the JN17 grains by increasing the accumulation of gliadin and LMW-GS, while the glutenin-to-gliadin ratio decreases significantly. This alteration in protein composition leads to deteriorated baking quality. Consequently, under salt stress, bread volume and bread score were reduced by 16.85% and 13.08%, respectively (Figure 2G–J).

### 2.3. Untargeted Metabolomic Analysis

**Figure 3:**
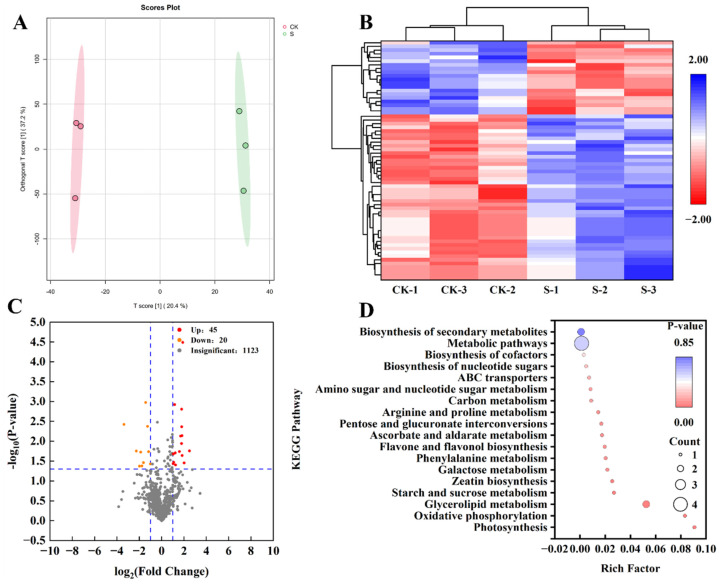
Metabolomic analysis of S compared to CK in winter wheat. (**A**): Orthogonal projections to latent structures-discriminate analysis (OPLS-DA) of metabolites. (**B**): Clustering heat map of DEMs. (**C**): Volcano of the DEMs. The horizontal blue dashed line in the figure represents the statistical significance threshold. Points above the horizontal line have *p* < 0.05, and points below the horizontal line have *p* > 0.05. The vertical dashed lines represent the fold change of differences. Points outside the right vertical line represent metabolites with significantly up—regulated expression, points outside the left vertical line represent metabolites with significantly down—regulated expression, and points between the two lines represent no significant difference. (**D**): KEGG pathway analysis of DEMs.

To analyze the metabolites of strong-gluten wheat JN17 under salt stress, a comprehensive untargeted metabolome analysis was conducted using an LC-MS system. A total of 65 metabolites were identified across all samples (Figure 3C). These metabolites were clearly separated into two distinct groups based on Orthogonal projections to latent structures-discriminate analysis (OPLS-DA) (Figure 3A), indicating significant differences in metabolite profiles of strong-gluten wheat JN17 under salt stress.

The volcano map provides a comprehensive visualization of the spatial distribution of DEMs, as depicted in Figure 3C. For the comparison between CK and S, an exhaustive analysis revealed 65 DEMs, with 45 exhibiting upregulation and 20 displaying downregulation, all adhering to the criteria of |Fold change| ≥ 2 and VIP ≥ 1.

A comprehensive KEGG enrichment pathway analysis was undertaken to precisely identify the pivotal metabolic pathways that underlying the response of winter wheat to NaCl stress. To gain deeper insight into the metabolic disparities across the comparison group, we conducted enrichment and topological analyses, selecting the top 18 most enriched pathways in each group for further examination (Figure 3D). Remarkably, several pathways emerged as consistently significant across the CK and S, including “Metabolic pathways”, “Biosynthesis of secondary metabolites”, “Arginine and proline metabolism”, “Starch and sucrose metabolism”, and “Phenylalanine metabolism”. These findings provide valuable insight into the complex metabolic adaptations of winter wheat in response to salt stress.

### 2.4. DIA-Based Proteomic Analysis

**Figure 4:**
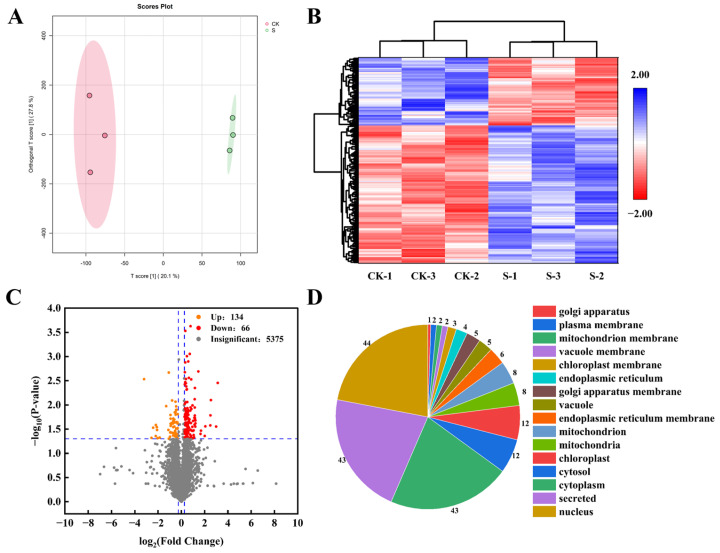
Proteomics analysis of S compared to CK in strong-gluten winter wheat. (**A**): OPLS-DA of proteins. (**B**): Clustering heat map of DAPs. (**C**): Volcano of the DAPs. The horizontal blue dashed line in the figure represents the statistical significance threshold. Points above the horizontal line have *p* < 0.05, and points below the horizontal line have *p* > 0.05. The vertical dashed lines represent the fold change of differences. Points outside the right vertical line represent metabolites with significantly up—regulated expression, points outside the left vertical line represent metabolites with significantly down—regulated expression, and points between the two lines represent no significant difference (**D**): Subcellular localization of DAPs. The numbers represent the DAPs located at this position.

To better understand the effects of salt stress on the winter wheat protein, a DIA-high-quality spectral libraries strategy was performed. A total of 5375 proteins were obtained (Figure 4C). As with the metabolomic analysis, the OPLS-DA of proteomic detected an obvious separation (Figure 4A). Compared with CK, 200 DAPs showed significant changes in salt stress, of which 134 proteins were upregulated, and 66 proteins were downregulated (Figure 4A). These proteins were mainly involved in the nucleus, secreted, and cytoplasm (Figure 4D).

### 2.5. Transcriptomic Analysis

**Figure 5:**
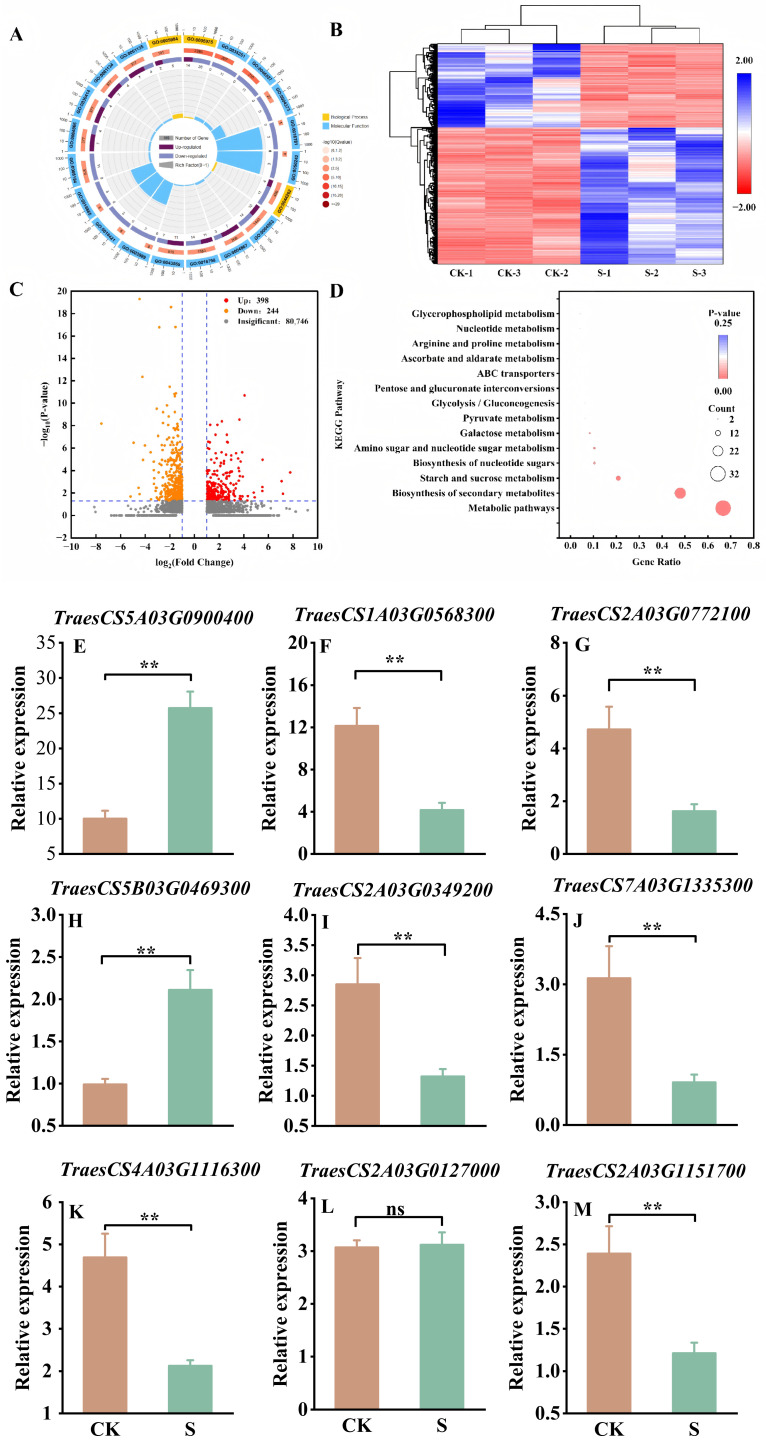
Transcriptomic analysis and RT-qPCR validation of differentially expressed genes of S compared to CK in strong-gluten wheat. (**A**): Top 20 GO enrichment circle diagram of DEGs. (**B**): Clustering heat map of DEGs. (**C**): Volcano of the DEGs. (**D**): KEGG pathway analysis of DEGs. (**E**–**M**) represent the relative expression levels of a single gene. *TraesCS5A03G0900400*: Ornithine aminotransferase. *TraesCS1A03G0568300*: β-amylase 3. *TraesCS2A03G0772100*: 1,4-α-glucan-branching enzyme 2. *TraesCS5B03G0469300*: Glucose-1-phosphate adenylyltransferase small subunit 1. *TraesCS2A03G0349200*: Sucrose synthase 2. *TraesCS7A03G1335300*: 1,4-α-glucan-branching enzyme. *TraesCS4A03G1116300*: Sucrose synthase 1. *TraesCS2A03G1151700*: 2-oxoglutarate 3-dioxygenase. *TraesCS2A03G0127000*: Probable amidase At4g34880. ** represents *p* < 0.01, ns represents not significant.

A total of 81,388 clean readings were obtained, and pairwise comparison of CK vs. S identified 642 (398 upregulated and 244 downregulated) DEGs (|Fold change| > 2 and *p* < 0.05) (Figure 5C).

The Gene Ontology (GO) enrichment analysis offered a refined categorization of the DEGs into three distinct yet interconnected groups: cellular components (CCs), biological processes (BPs), and molecular functions (MFs). The top 20 enrichment circle diagrams of the DEGs are presented in Figure 5A. There were three items enriched in BP, among which was GO:0005984 (mannose metabolic process). To further validate the reliability of the identified DEGs in response to salt stress, we selected nine differential genes from four key metabolic pathways: the arginine and proline metabolic pathway, starch and sucrose metabolic pathway, flavonoid biosynthesis pathway, and tryptophan metabolic pathway for validation of their expression profiles in response to salt stress in developing grains, except for the gene *TraesCS2A03G0127000* (which showed no significant difference); the expression patterns of differentially expressed genes (DEGs) obtained from RNA-seq and RT-qPCR were consistent, thus supporting the reliability of the data (Table S1, Figure 5E–M).

## 3. Discussion

### 3.1. Effects of Salt Stress on Yield and Yield Components of Strong-Gluten Wheat

Patwa et al. (2024) found that salt stress significantly reduced the spike number, grain number per spike, and 1000-grain weight of wheat by inhibiting the occurrence of wheat tillering, reducing the seed-setting rate, and suppressing the grain filling process, thereby ultimately leading to a decline in yield [17]. This study showed that salt stress significantly reduced the grain yield of JN17 (Figure 1D). Specifically, under salt stress, the spike number, grain number per spike, and 1000-grain weight of JN17 were all inhibited, with the 1000-grain weight showing the largest decrease amplitude, which is consistent with previous research findings [22] (Figure 1A–C). Specifically, salt stress increases the content of propanediol in leaves, destroys the chloroplast structure, reduces the leaf photosynthetic rate, accelerates leaf senescence, decreases the transport of photosynthates to grains, and weakens the photosynthetic assimilation capacity. These effects are the key physiological mechanisms for the decrease in 1000-grain weight [23]. Furthermore, osmotic stress and ionic toxicity caused by salt stress (such as the accumulation of Na^+^ in leaves) can interfere with the synthesis of starch and protein in grains, further exacerbating the loss of grain weight [17]. The significant reduction in grain weight of JN17 provides an explanation for the concentration of the increase in relative protein content.

### 3.2. Effects of Salt Stress on Protein Content and Composition in Strong-Gluten Wheat

The results of this study indicated that salt stress significantly increased the protein content of JN17 grains. From the perspective of protein composition, the increase in grain protein content was mainly attributed to the elevated levels of gliadin and glutenin, with gliadin showing a greater increase than glutenin, which led to a decrease in the glutenin-to-gliadin ratio under salt stress. This study’s findings are consistent with previous research demonstrating that salt stress increases both grain protein content and gliadin levels in wheat. However, our results differ from earlier studies that reported a decrease in glutenin content under salt stress, which may be attributable to differences in salt concentration and wheat cultivar [24].

Further analysis of the glutenin composition in grains under salt stress revealed that the increase in glutenin content was primarily due to the rise in low molecular weight glutenin subunits (LMW-GS), while there was no significant change in high molecular weight glutenin subunits (HMW-GS), resulting in a decline in the HMW-GS/LMW-GS ratio. A decrease in the glutenin-to-gliadin ratio may directly weaken the strength and elasticity of the gluten network. HMW-GSs are the core components for cross-linking in the gluten network; insufficient content of HMW-GS reduces the gas-holding capacity and extensibility of dough, leading to smaller bread volume and loose texture [25]. Although a significant increase in gliadin can enhance gluten extensibility, its synergistic effect with LMW-GS may be weakened due to the insufficient HMW-GS, forming a gluten structure characterized by “high extensibility but low elasticity,” which further affects bread volume and texture [25,26]. In addition, the salt stress-induced increase in the activities of antioxidant enzymes (e.g., SOD and POD) may protect the integrity of glutenin macropolymer (GMP) by alleviating oxidative damage; however, the energy dependence of HMW-GS synthesis (which requires photosynthate support) may be restricted due to the inhibition of photosynthesis by salt, resulting in no significant increase in its proportion [27]. Therefore, under the conditions of this experiment, although salt stress increased the protein content of JN17, the decrease in the ratio of HMW-GS to LMW-GS and the glutenin-to-gliadin ratio, because of the significant increase in the contents of gliadin and LMW-GS, led to reductions in the elasticity, toughness, and strength of the gluten in JN17 (Figure 2A–D). Ultimately, this resulted in an increase in the grain protein content of JN17, accompanied by a decrease in its protein quality (Figure 1E and Figure 2A).

### 3.3. Multi-Omics Joint Analysis

**Figure 6:**
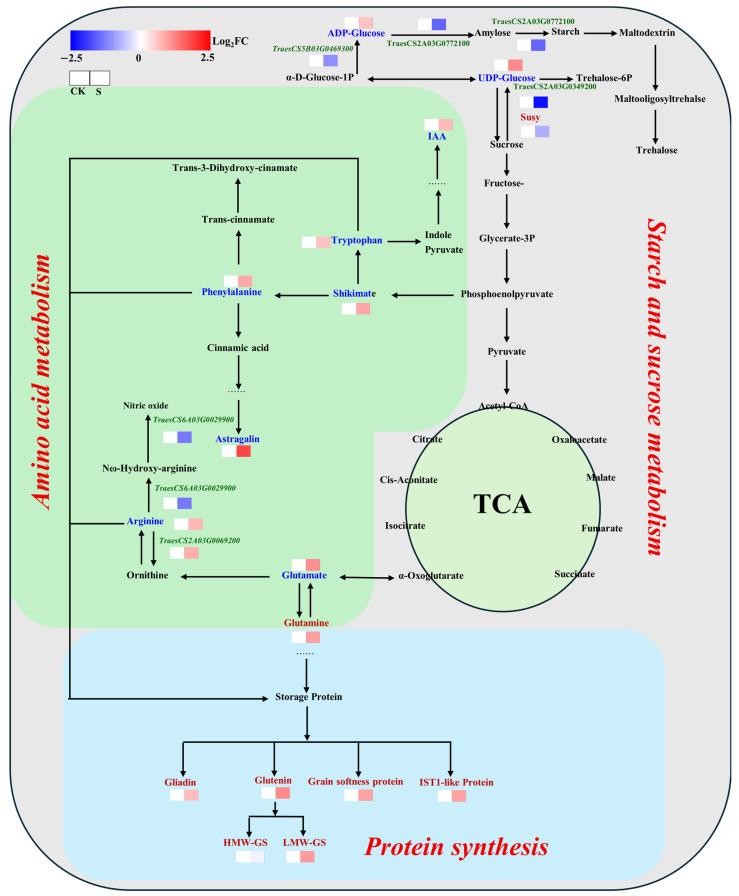
Analysis of key metabolic pathways involving differential metabolites, proteins, and genes in strong-gluten wheat grains under salt stress. Blue font represents differential expression metabolites, red font represents differential expression proteins, and green font represents differential expression genes. In the two panels, the left panel displays the Log_2_FC of metabolites/proteins/genes in the control group, while the right panel presents their Log_2_FC under salt stress treatment. The transparent colors in the left panel correspond to the Log_2_FC values of the control group, whereas the colors in the right panel reflect the Log_2_FC values under salt stress. Redder hues indicate a greater upregulation of metabolites/proteins/genes under salt stress relative to the control group, while bluer hues denote a greater downregulation.

Integrated multi-omics analysis, combining physiological biochemistry, metabolomics, proteomics, and transcriptomics, systematically reveals the molecular mechanisms by which salt stress affects quality formation in JN17 through synergistic regulation of carbon and nitrogen metabolic networks. The research indicated that salt stress reconstructs the allocation balance of carbon and nitrogen metabolic fluxes: salt stress suppresses the carbon and nitrogen metabolism processes in grains, with a significantly stronger inhibitory effect on carbon metabolism than on nitrogen metabolism, resulting in an increase in grain protein content. Meanwhile, the elevated levels of some amino acids are redirected toward the synthesis of osmotic adjustment substances, further exacerbating the reprogramming of metabolic flux. Ultimately, this leads to a paradoxical phenotype where grain protein content increases while processing quality decreases (Figure 6).

#### 3.3.1. Multi-Level Suppression Mechanism of Starch Synthesis Pathway

Salt stress reduces the transport and absorption of nutrients and sucrose in JN17 through transcriptional regulation and enzyme activity inhibition. Transcriptome data showed that the expression of key genes involved in sucrose degradation (e.g., *TraesCS2A03G0349200*-LOC123187938, sucrose synthase 2) (down 80.20%) and core starch synthesis genes (e.g., *TraesCS5B03G0469300*-LOC123111653glucose-1-phosphate adenylyltransferase small subunit 1, *TraesCS2A03G0772100*-LOC123189301, 1,4-alpha-glucan-branching enzyme 2) (down 52.87% and 64.59%, respectively) were significantly downregulated. This observation correlates with the sucrose accumulation phenomenon noted in our metabolomic data (Figure 6). Notably, this regulatory pattern is highly consistent with reports under other stress conditions like high temperature and drought [28], suggesting that crop responses to different adversities may have some common metabolic regulation pathways.

Meanwhile, research indicated that salt stress disrupts chloroplast membrane system integrity via Na^+^/K^+^ imbalance, leading to inactivation of PSII reaction centers and reduced efficiency of the photosynthetic electron transport chain. Concurrently, it induces a burst of reactive oxygen species (ROS), triggering thylakoid membrane lipid peroxidation and significantly inhibiting RuBisCO activity and photosynthetic carbon assimilation capacity [29]. Next, the export of photosynthetic products is impeded: salt stress decreases the activity of sucrose synthase (SPS) in flag leaves, reducing sucrose synthesis [30]. Ultimately, at the grain level, salt stress damages amyloplast membrane structure and suppresses the activity of key starch synthesis enzymes. Additionally, it induces ClpP2-mediated degradation of starch synthases, resulting in abnormal chain length distribution of amylopectin and blocked accumulation of amylose, thereby inhibiting starch synthesis and transport in grains [31]. This provides new insights for deepening our future research.

Although metabolomic analysis detected significant accumulation of starch synthesis precursors ADP-glucose and UDP-glucose in the grains, these precursors were not efficiently utilized for starch synthesis. Therefore, the grain carbon metabolism is more severely affected by salt stress, thereby relatively enhancing the metabolic synthesis of certain amino acids.

#### 3.3.2. Stress-Induced Remodeling of Amino Acid Metabolic Network

Amino acids, as the core precursors for wheat protein synthesis, directly affect the composition, content, and quality formation of wheat grain proteins through their types, proportions, and metabolic dynamics [32]. Arginine, tryptophan, and phenylalanine, as essential amino acids, play crucial roles in glutenin synthesis and the proper folding of HMW-GS [33,34]. Metabolic profiling analysis revealed an interesting phenomenon: under salt stress, the synthesis of amino acid precursors like glutamic acid and shikimate significantly enhanced, providing ample carbon skeletons for the accumulation of arginine, phenylalanine, and tryptophan [35]. However, transcriptome data indicated that these amino acids were not entirely used for storage protein synthesis but were preferentially directed towards stress metabolism pathways. Specifically, the expression of key genes in the arginine metabolic pathway (e.g., *TraesCS6A03G0029900*-LOC123131611, putative nitric oxide synthase, *TraesCS2A03G0069200*-LOC123187054, arginase 1, mitochondrial-like) (up 144.33% and 74.17%) was significantly upregulated, channeling arginine towards the synthesis of osmoprotectants like N ω-hydroxy-L-arginine and ornithine (Figure 6).

More notably, the tryptophan metabolic pathway exhibited a distinct characteristic: on the one hand, the expression of genes related to tryptophan synthesis was enhanced; on the other hand, its downstream auxin biosynthesis pathway was significantly activated (see Figure 6), leading to the utilization of tryptophan for auxin synthesis. This finding is consistent with the study by Jing et al. (2023) [36]. It also indicated that plants remodel amino acid metabolism under salt stress to balance growth and stress resistance demands, resulting in the diversion of HMW-GS synthesis precursors toward the stress-resistant direction, which ultimately leads to no significant change in HMW-GS content.

#### 3.3.3. Regulatory Imbalance in Protein Assembly Process

Proteomic analysis provided direct evidence for understanding the quality deterioration mechanism. Although the accumulation of gliadin and LMW-GS increased by 24.76% and 19.28%, respectively, under salt stress, the content of HMW-GS—essential for forming a high-quality gluten network—increased only marginally by 2.16%, not reaching statistical significance (Figure 2B,D,E). This imbalance in protein component proportions directly led to a 14.35% decrease in the glutenin-to-gliadin ratio, severely affecting the elasticity and extensibility of the gluten network (Figure 2F).

In-depth analysis suggested that the limited synthesis of HMW-GS might be closely related to two factors: firstly, salt stress reprograms the carbon–nitrogen metabolic network, diverting key amino acids (such as arginine and tryptophan) originally intended for HMW-GS synthesis toward stress metabolism pathways (e.g., osmolyte and hormone biosynthesis), leading to a shortage of substrates for HMW-GS biosynthesis. Secondly, proteomic data further indicated that salt stress may disrupt correct protein folding and assembly through post-translational modifications (PTMs). For instance, recent studies reported significant lysine crotonylation (Kcr) modifications in wheat chloroplast proteins under salt stress; such modifications influence protein stability and function by altering surface charge distribution (e.g., Kcr at the K367 site of TaFBA6 enhances structural stability), and similar mechanisms may affect the assembly of storage proteins, resulting in reduced HMW-GS polymerization efficiency [37,38]. These findings provide new perspectives for understanding the molecular mechanism of protein quality formation under salt stress.

In summary, multi-omics analysis clearly elucidates the mechanism of quality formation in JN17 under salt stress: the stress signal inhibits starch–sucrose metabolism and reprograms the carbon–nitrogen allocation network. This not only reduces grain weight but also causes an imbalance in the proportions of protein components, ultimately manifesting as the phenomenon of increased content but decreased quality.

### 3.4. Impact of Salt Stress on Commercial Value of Strong-Gluten Wheat

In the grain trade, the pricing of wheat depends not only on yield indicators such as kernel weight but also closely on quality parameters [1]. Experimental results showed that although salt stress increased the protein content of strong-gluten wheat Jinan 17 by 13.82%, the bread volume and bread score decreased by 16.85% and 13.08%, respectively (Figure 2A,G,H), ultimately leading to a deterioration in baking quality. This deterioration was attributed to an increase in gliadin content and LMW-GS content, along with no significant change in HMW-GS content, which resulted in a reduced glutenin-to-gliadin ratio and HMW-GS/LMW-GS ratio. This quality decline directly reduces the commercial appeal of wheat, as the baking industry highly relies on specific gluten network structures to ensure product performance [11].

A study indicated that HMW-GS content is an important parameter affecting the quality of strong-gluten wheat and a key factor in trade pricing [12]. Multi-omics analysis further explains that salt stress, by inhibiting starch synthesis and altering amino acid metabolism, leads to no significant change in HMW-GS content (Figure 2F), forming a gluten network with high extensibility but low elasticity. Such quality variation can lower the premium pricing ability of wheat in the market. Therefore, under salt stress conditions, although strong-gluten wheat may maintain high protein content, quality deterioration often leads to price discounts, ultimately affecting farmers’ economic returns.

## 4. Materials and Methods

### 4.1. Experimental Design

A pot experiment was conducted during the winter wheat growing seasons from 2024 to 2025 at the Science and Technology Backyard of Shandong Daiyue Wheat Cement Pond (36°00′ N, 117°01′ E), Daiyue District, Tai’ an City, Shandong Province. The average temperature during the wheat growing seasons from October 2024 to June 2025 was 10.09 °C, with a total precipitation of 162.60 mm across the entire growth period.

In this experiment, the strong-gluten winter wheat variety, Jinan 17 (JN17), was used as the test material. Two treatments were set up: non-stress treatment (CK) and salt stress treatment (with a NaCl concentration of 2.8‰, S). There were 300 pots for each treatment, with a total of 600 pots. To prevent the leakage of salt solution, the pot experiment was carried out using non-porous polyvinyl chloride plastic pots (with a bottom diameter of 23.5 cm and a height of 26.0 cm). Each pot was filled with 14 kg of well-mixed soil. For the S treatment, the amount of NaCl required for each pot was calculated based on the amount of soil filled. The mass of NaCl for each pot was 39.2 g. The corresponding amount of NaCl was dissolved in 150 mL of deionized water and mixed with the soil before potting. For the CK treatment, only 150 mL of deionized water was added. Each pot was applied with 2.5 g of urea (containing 46% N), and the ratio of base fertilizer to top-dressing (at the elongation stage) was 4:6. At the same time, 10 g of superphosphate per pot (containing 12% P_2_O_5_) and 2 g of potassium chloride per pot (containing 60% K_2_O) were applied at one time before potting. Twelve seeds were evenly planted in each pot, and after emergence, the seedlings were thinned to 8 per pot. The chemical properties of the soil were as follows: organic matter 12.85 g/kg, total nitrogen 1.30 g/kg, alkaline-hydrolysable nitrogen 75.32 mg/kg, available phosphorus 17.03 mg/kg, and available potassium 133.78 mg/kg. The field growth status of wheat is shown in Figure S1.

### 4.2. Indicators and Their Determination Methods

#### 4.2.1. Yield and Yield Components

For each treatment, 70 pots of plants with uniform growth were selected at the maturity stage to investigate the number of panicles per pot, panicles per plant, and grains per panicle. The plants were then manually harvested and threshed. After air-drying, the 1000-grain weight was measured. Each treatment was replicated three times.

#### 4.2.2. Protein Content and Components

The grain nitrogen content of wheat at maturity was measured using the Kjeldahl method, and the protein content of grains was calculated by multiplying the nitrogen content of grains by 5.7. Grain protein yield is the product of grain yield and grain protein content. The contents of gliadin, HMW-GS, LMW-GS, and glutenin in grains were determined using a high-performance liquid chromatograph [39]. The glutenin-to-gliadin ratio refers to the content ratio of glutenin to gliadin in grains.

#### 4.2.3. Bread Preparation and Quality Assessment

Bread was prepared according to the Chinese National Standard (GB/T 35869-2018) [40] with modifications. The dough formulation consisted of flour (100 g, 14% moisture basis), salt (1.5 g), sugar (6 g), nonfat dry milk (4 g), and shortening (3 g). Salt and sugar were pre-dissolved in 50 mL of water (≈40 °C). Flour, shortening, nonfat dry milk, and yeast were placed in a dough mixer, followed by the addition of 15 mL of water. Mixing was performed for 5 min using the dough development program until optimal dough consistency was achieved. The dough was proofed at 30 °C and 85% relative humidity for 90 min. During proofing, dough punching was performed using a sheeter at 55 and 80 min to degas. At 90 min, the dough was molded and placed in baking pans. After shaping, final proofing was conducted for 45 min, followed by baking in a preheated oven at 215 °C for 20 min. Bread volume was determined by the rapeseed displacement method [41]. Bread quality scoring followed the Chinese National Standard (GB/T 35869-2018) [40]. Three loaves of bread were produced for each treatment.

#### 4.2.4. Multi-Omics Analysis

For each treatment, 5 wheat plants with uniform growth were selected at 14 days after anthesis, and their samples were mixed and stored in a −80 °C ultra-low temperature freezer as omics submission samples. Three biological replicates were included for each treatment, resulting in a total of 30 pots of plants. When submitting the samples, the middle part grain of each spikelet was taken from the plants under liquid nitrogen conditions for submission. Three replicates were prepared for each treatment according to the sampling classification.

##### Metabolomic Analysis

The metabolite extraction and identification of wheat samples were done by the Smartgenomics Technology Institute (Tianjin, China). Fanelli et al. (2023) [42] described this standardized procedure and instrument in detail in the untargeted metabolome program.

The grain samples (20 mg ± 1 mg) were taken and lyophilized and mixed with beads and 1000 μL of extraction solution (MeOH: ACN: H_2_O, 2:2:1 (*v*/*v*)). The extraction solution contains deuterated internal standards. The mixed solutions were vortexed for 30 s. Then, the mixed samples were homogenized (35 Hz, 4 min) and sonicated for 5 min in a 4 °C water bath, and this step was repeated three times.

The samples were incubated for 1 h at −40 °C to precipitate proteins. Then, the samples were centrifuged at 12,000 rpm (RCF = 13,800× *g*, R = 8.6 cm) for 15 min at 4 °C. Transfer 400 μL of liquid to the well of a protein precipitation plate. Place the plate on the manifold. Apply vacuum, at 6 psi, for 120 s. Take the plate from the positive pressure device for analysis. The quality control sample was prepared by mixing an equal aliquot of the supernatant of the samples.

LC-MS/MS analyses were performed using an UHPLC system (Vanquish, Thermo Fisher Scientific, Waltham, MA, USA) with a Phenomenex Kinetex C18 (2.1 mm × 100 mm, 2.6 μm) coupled to an Orbitrap Exploris 120 mass spectrometer (Orbitrap MS, Thermo). The mobile phase A: 0.01% acetic acid in water; mobile phase B: IPA: ACN (1:1, *v*/*v*). Column temperature was 25 °C. The auto-sampler temperature was 4 °C, and the injection volume was 2 μL.

The Orbitrap Exploris 120 mass spectrometer was used for its ability to acquire MS/MS spectra on information-dependent acquisition mode in the control of the acquisition software (Xcalibur 4.4, Thermo). In this mode, the acquisition software continuously evaluates the full scan MS spectrum. The ESI source conditions were set as follows: sheath gas flow rate as 50 Arb, Aux gas flow rate as 15 Arb, capillary temperature 320 °C, Sweep Gas: 1 Arb, Vaporizer Temp: 350 °C, full MS resolution as 60,000, MS/MS resolution as 15,000, collision energy: SNCE 20/30/40, and spray voltage as 3.8 kV (positive) or −3.4 kV (negative).

##### Proteomics Analysis

The sample preparation encompassed protein extraction, bicinchoninic acid quantification, reduction/alkylation, and enzymatic digestion. Initially, 700 μL of phenol extraction buffer was added to the sample, followed by homogenization with steel beads. After adding Tris-buffered phenol (pH 8.0), the mixture was incubated at 4 °C for 40 min with intermittent shaking. The supernatant was collected by centrifugation (12,000 rpm, 4 °C, 10 min), and proteins were precipitated overnight at −20 °C using a 0.1 M ammonium acetate–methanol solution. The pellet was washed sequentially with chilled methanol and acetone, air-dried, and resuspended in RIPA lysis buffer. Ultrasonication (ice-water bath, 20 min) and centrifugation (12,000 rpm, 4 °C, 10 min) yielded the protein extract.

Protein concentration was determined via BCA assay: BCA working reagent (200 μL/well) and standards (BSA, 7-point curve) were mixed with 20 μL of the sample (diluted as needed), incubated at 37 °C for 30 min, and measured at 562 nm. For digestion, 40 μL of protein solution was acetone-precipitated (−40 °C, 4 h) and subjected to reduction (95 °C, 5 min) and alkylation. Trypsin digestion proceeded at 37 °C for 2 h and was terminated by quenching reagent. Peptides were desalted using a solid-phase extraction column: sequential washes with reagents D/E (100 μL × 2 each) removed salts, followed by elution with reagent F (100 μL × 2). The eluate was vacuum-concentrated, reconstituted in mobile phase A (supplemented with iRT for DIA), and quantified via NanoDrop, and equal amounts were loaded for LC-MS/MS analysis.

For each sample, 200 ng of total peptides were separated and analyzed with a nano UPLC (nanoElute2) coupled to a timsTOF Pro2 instrument (Bruker, Karlsruhe, Germany) with a nano electrospray ion source. Separation was performed using a reversed-phase column (PePSep C18, 1.9 μ, 75 μ × 15 cm, Bruker, Karlsruhe, Germany). Mobile phases were H_2_O with 0.1% FA (phase A) and CAN with 0.1% FA (phase B). The mass spectrometer adopts DIA PaSEF mode for DIA data acquisition, and the scanning range is from 350 to 1250 *m*/*z*. During PASEF MS/MS scanning, the impact energy increases linearly with ion mobility, from 20 eV (1/K0 = 0.85 Vs/cm^2^) to 59 eV (1/K0 = 1.30 Vs/cm^2^).

Vendor’s raw MS files were processed using Spectronaut software (19.0.240604.62635) and the built-in Pulsar search engine. MS spectra lists were searched against their species-level UniProt FASTA databases, with Carbamidomethyl as a fixed modification and Oxidation and Acetyl as variable modifications. Trypsin was used as proteases. A maximum of 2 missed cleavage(s) was allowed. The false discovery rate was set to 0.01 for both PSM and peptide levels. Peptide identification was performed with an initial precursor mass deviation of up to 20 ppm and a fragment mass deviation of 20 ppm. All the other parameters were reserved as default.

##### Transcriptome Sequencing

The total RNA from tissues or cells was extracted using the RNA prep Pure Plant Plus Kit (TIANGEN, Beijing, China). The RNA samples then underwent stringent quality control, primarily through the Agilent 2100 Bioanalyzer (Santa Clara, CA, USA), which precisely detects RNA integrity. After the RNA quality was confirmed, the Hieff NGS^®^ Ultima Dual-mode RNA Library Prep Kit (Premixed version) (Yeasen, Shanghai, China) was used for transcriptome library construction, strictly following the instructions regarding recommended reagents and consumables. Differential expression analysis between the two comparative combinations was performed using DESeq2 data analysis method [43]. Genes found to have adjusted *p* < 0.05 by DESeq2 were assigned as differentially expressed. Differential gene enrichment analysis used cluster Profiler software v3.0 to perform Gene Ontology (GO) functional enrichment analysis and Kyoto Encyclopedia of Genes and Genomes (KEGG) pathway enrichment analysis on the differential gene sets [44]. padj less than 0.05 was used as the threshold of significant enrichment for GO functional enrichment and KEGG pathway enrichment.

#### 4.2.5. RT-qPCR Analysis

Total RNA was extracted from fresh middle-segment grain samples of 14-day-old wheat spikes using the RNA-easy Isolation Reagent (R701, Vazyme, Nanjing, China). A liquid nitrogen-prechilled mortar was used to grind the fresh grain samples into a fine powder with liquid nitrogen. Approximately 0.1 g of the powdered sample was transferred to a 1.5 mL EP tube, followed by the addition of 0.5 mL of RNA-casy reagent. Subsequent steps were performed according to the kit instructions. After extraction, the RNA was dissolved in 50 μL of DEPC-treated water, and its purity and concentration were measured using a NanoDrop 2000 spectrophotometer (Thermo Scientific, Waltham, MA, USA), with the OD260/OD280 ratio maintained within the range of 1.8–2.2.

First-strand cDNA synthesis was carried out using the HiScript II RT SuperMix for qPCR kit (R323, Vazyme, Nanjing, China). Quantitative real-time PCR (qPCR) was performed using the ChamQ SYBR qPCR Master Mix kit (Q311, Vazyme, Nanjing, China) and gene-specific primers, with the LightCycler 96 real-time PCR system (Roche, Basel, Switzerland). The PCR reaction program consisted of an initial denaturation at 95 °C for 30 s, 40 cycles of denaturation at 95 °C for 10 s, annealing at 60 °C for 30 s, followed by a final step at 95 °C for 15 s, 60 °C for 60 s, and 95 °C for 15 s. Three biological replicates and three technical replicates were included for each sample.

Gene expression data were standardized using the CFX96 real-time quantitative system (Bio-Rad, Hercules, American). The relative quantification of gene expression was calculated using the 2^−ΔΔCt^ method, where ΔΔCt = (Ct_target gene_ − Ct_Actin_) treatment − (Ct_target gene_ − Ct_Actin_) control, with Ct representing the cycle threshold at which the fluorescence signal of the target gene crosses the predefined threshold. Gene primers are listed in Supplementary Information Table S1.

### 4.3. Statistical Analysis

The yield and quality-related data were analyzed by one-way ANOVA via SPSS 26.0 (IBM, New York, NY, USA). The annotations and classification of the differential expression genes (DEGs) and differential expression metabolites (DEMs) were based on the KEGG. The annotations of the differential abundant proteins (DAPs) were based on the databases of GO. Bar graphs, volcano graphs, heatmaps, KEGG pathway enrichment graphs, and pie graphs were plotted via Origin 2024 (Origin Lab Corporation, North Hampton, MA, USA). GO enrichment circle diagram of differential expression genes was plotted via Omics share (https://www.omicshare.com/).

## 5. Conclusions

In summary, compared with the control, salt stress reduced the spike number, grains per spike, and 1000-grain weight of the strong-gluten wheat variety Jinan 17, with the most significant yield reduction attributed to decreased 1000-grain weight. Under salt stress, the protein components of JN17 exhibited increased gliadin, glutenin, and LMW-GS content, reduced glutenin-to-gliadin ratio, and unchanged HMW-GS content. These findings indicate that salt stress decreased the bread-making quality of JN17 by significantly enhancing LMW-GS and gliadin accumulation. Multi-omics analysis reveals that salt stress, by altering metabolite accumulation and gene expression, inhibits carbon metabolism to a greater extent than nitrogen metabolism, thereby indirectly promoting the biosynthesis of amino acids and their derivatives. This study reveals the molecular mechanisms of salt stress-induced protein quality changes in JN17 and identifies candidate genes for further improving protein quality under saline conditions.

## Data Availability

The original contributions presented in this study are included in the article/Supplementary Materials. Further inquiries can be directed to the corresponding authors.

## Supplementary Materials

The following supporting information can be downloaded at: https://www.mdpi.com/article/10.3390/ijms27073013/s1.
