# Supplementary material for: Multi-Omics Reveals Salt Stress Effects on Quality Formation of Strong-Gluten Wheat
Source: Int J Mol Sci. 2026 Mar 26;27(7):3013. doi: 10.3390/ijms27073013 (PMC13073276; doi:10.3390/ijms27073013)
Supplement: Supplementary file 1 [file ijms-27-03013-s001.zip › Supplemental information.pdf]

Multi-omics reveals salt stress effects on quality formation of strong-gluten wheat

Wei Zhou[1], Jianchao Zheng[2], Yonggang Zhao[3], Shikui Li[2], Hongxia Zhang[3], Xiang Li[4], Chuan Zhong[1]*, Xinglong Dai[1]*

[1] College of Agriculture, Shandong Agricultural University / State Key Laboratory of Wheat Improvement, Tai'an 271018, China

[2] Agricultural Science Research Institute of the Twelves Division of Xinjiang Production and Construction Crops, Urumqi 830500, China

[3] Agricultural Science Research Institute of the Seventh Division of Xinjiang Production and Construction Crops, Yili 833200, China

[4] College of Life Sciences, Shandong Agricultural University, Tai'an 271018, China

*Corresponding author.

E-mail addresses: Zhongchuan@sdau.edu.cn (C. Zhong); adaisdny@163.com (X L. Dai).

Table. S1 Primers used for RT-qPCR validation

Genes	Forwards (5'→3')	Reverse (5'→3')
TraesCS5A03G0900400	CCCATTTCAATCAGTCCCGA	ATCGCAGACGGGTGTTTTTG
TraesCS1A03G0568300	TCGGTGACTCCTGCAACATC	CACGATGTCCGGGTCGG
TraesCS2A03G0772100	GCACTTCACTTCTGACTGCC	GTGCTAGCAACAACAGCGTG
TraesCS5B03G0469300	CGCCATATATATAAACCAGGTTGGC	CATGAGAAACCAGCCGTGGA
TraesCS2A03G0349200	CCAGTTCGCCTCGCGTTT	TTGCTGTCCTCAAACCTTGC
TraesCS7A03G1335300	AGGAACAAAGATCAGACGACTCC	CGAACGTGCTTGTGGAACTT
TraesCS4A03G1116300	TAAATACCCTGCCTCGCCGT	CAAGCCTAGTCTGCTTGGCA
TraesCS2A03G1151700	CGACCACGGCCACTTCAT	GCTCTCCCCGTTCACCAC
TraesCS2A03G0127000	ACCATCCTGTGGAGGAGAGG	CTCCTTCAGGCGACGGATTG

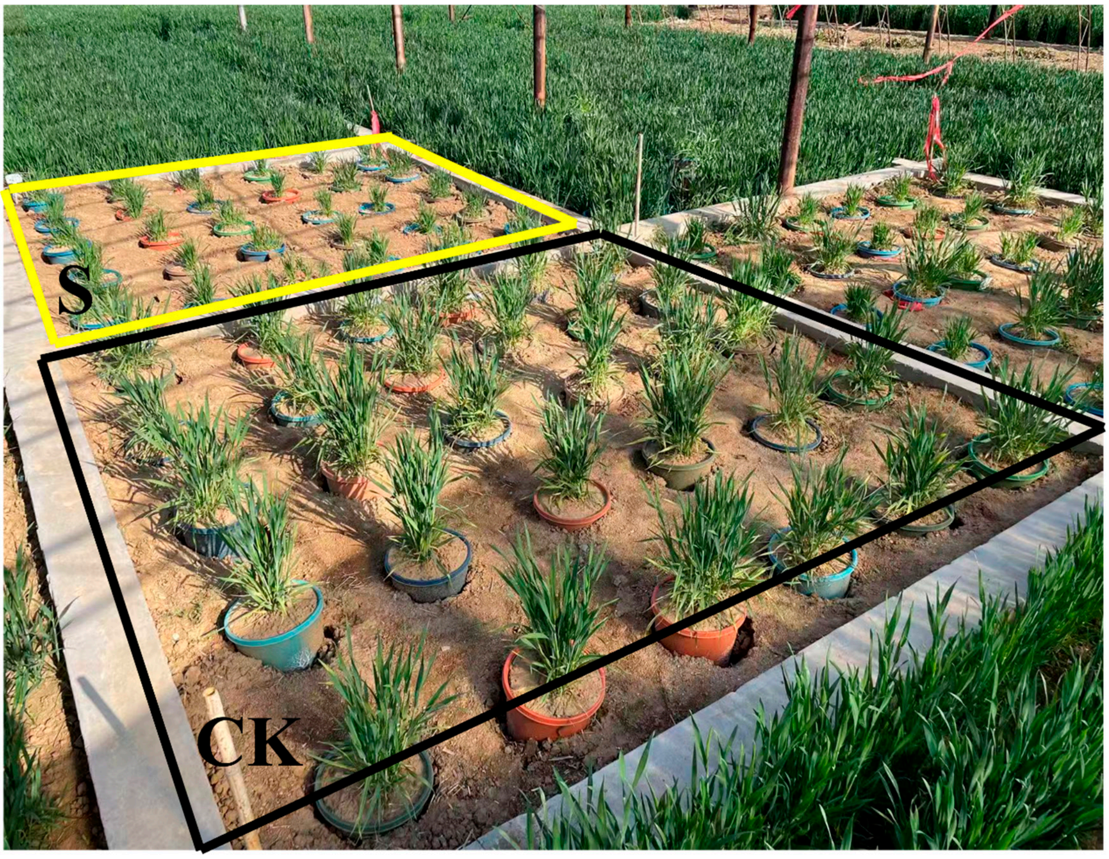

Figure. S1 Wheat growth status under non-salt stress and salt stress conditions .CK: Control group, S: NaCl concentration of 2.8 ‰.
